# Decreased GTPase activity of K- *ras* mutants deriving from human functional adrenocortical tumours

**DOI:** 10.1054/bjoc.1999.1039

**Published:** 2000-02-01

**Authors:** S-R Lin, C-H Hsu, J-H Tsai, J-Y Wang, T-J Hsieh, C-H Wu

**Affiliations:** Department of Internal Medicine and Clinical Pathology, Kaohsiung Medical College, No 100, Shih-Chuan 1st Rd, Kaohsiung, 80317, Taiwan

**Keywords:** GTPase, K-*ras* mutants, functional adrenocortical tumours

## Abstract

Our previous studies have shown that seven out of 15 patients with adrenocortical tumours contained K- *ras* gene mutation. In addition, the mutation type was a multiple-site mutation, and the hot spots were located at codons 15, 16, 18 and 31, which were different from those reported before (codons 12, 13 and 61). To understand whether the mutation hot spots in human adrenocortical tumours were associated with activation of K- *Ras* oncogene and the alterations of its biocharacteristics, mutant K- *Ras* genes were cloned from tumour tissues and then constructed with expression vector pBKCMV. Mutant K- *Ras* genes were expressed at high levels in *Escherichia coli* and the resultant K- *Ras* proteins were shown to be functional with respect to their well-known specific, high-affinity, GDP/GTP binding. The purified K- *Ras* protein from *E. coli* were then measured for their intrinsic GTPase activity and the GTPase activity in the presence of GTPase-activating protein for Ras. The results showed that the wild-type cellular K- *Ras* protein (p21BN) exhibits about ten times higher intrinsic GTPase activity than the activated protein (p21BM3) encoded by mutant K- *Ras* gene, which mutated at codon 60. With regards to the codon 15, 16, 18 and 31 mutant K- *Ras* proteins (p21BM2), the GTPase activity in the presence of GAP is much lower than that of the normal K- *Ras* protein, whereas the intrinsic GTPase activity is nearly the same as that of the normal K- *Ras* protein. These results indicated that mutations at these hot spots of K- *Ras* gene were indeed activated K- *Ras* oncogene in adrenocortical tumours; however, their association with tumors needs further experiments to prove. © 2000 Cancer ResearchCampaign


*British Journal of Cancer* (2000) **82**(5), 1035–1040
© 2000 Cancer Research Campaign
DOI: 10.1054/ bjoc.1999.1039, available online at http://www.idealibrary.com on IDEAL®

### Decreased GTPase activity of K-*ras* mutants deriving from human functional adrenocortical tumours

**S-R Lin[2], C-H Hsu[2], J-H Tsai[1], J-Y Wang[2], T-J Hsieh[2] and C-H Wu[2]**

Department of [1]Internal Medicine and [2]Clinical Pathology, Kaohsiung Medical College, No 100, Shih-Chuan 1st Rd, Kaohsiung, 80317, Taiwan

**Summary** Our previous studies have shown that seven out of 15 patients with adrenocortical tumours contained K-*ras* gene mutation. In addition, the mutation type was a multiple-site mutation, and the hot spots were located at codons 15, 16, 18 and 31, which were different from those reported before (codons 12, 13 and 61). To understand whether the mutation hot spots in human adrenocortical tumours were associated with activation of K-*Ras* oncogene and the alterations of its biocharacteristics, mutant K-*Ras* genes were cloned from tumour tissues and then constructed with expression vector pBKCMV. Mutant K-*Ras* genes were expressed at high levels in *Escherichia coli* and the resultant K-*Ras* proteins were shown to be functional with respect to their well-known specific, high-affinity, GDP/GTP binding. The purified K-*Ras* protein from *E. coli* were then measured for their intrinsic GTPase activity and the GTPase activity in the presence of GTPase-activating protein for Ras. The results showed that the wild-type cellular K-*Ras* protein (p21BN) exhibits about ten times higher intrinsic GTPase activity than the activated protein (p21BM3) encoded by mutant K-*Ras* gene, which mutated at codon 60. With regards to the codon 15, 16, 18 and 31 mutant K-*Ras* proteins (p21BM2), the GTPase activity in the presence of GAP is much lower than that of the normal K-*Ras* protein, whereas the intrinsic GTPase activity is nearly the same as that of the normal K-*Ras* protein. These results indicated that mutations at these hot spots of K-*Ras* gene were indeed activated K-*Ras* oncogene in adrenocortical tumours; however, their association with tumors needs further experiments to prove. © 2000 Cancer Research Campaign

**Keywords**: GTPase; K-*ras* mutants; functional adrenocortical tumours

Among the studies of oncogenes in human tumours, activation of *ras* oncogene has been found to be the most common event in various cancers including bladder cancers, breast cancers, colon cancers, kidney cancers, liver cancers, lung cancers, ovarian cancers, pancreatic cancers and gastric cancers (Yamashita et al, 1995; Muleahy et al, 1998; Caduff et al, 1999). In addition, activation of K-*ras* gene was found in more than 40% of colon cancer, pancreatic cancers and lung cancers (Anker et al, 1997; Gao et al, 1997). Our previous study has also showed that mutations of the K-*ras* gene were found in 55% of adrenocortical tumours and the mutation hot spots were focused on codons 15, 16, 18 and 31 (Lin et al, 1998), which were different from those (codons 12, 13 and 61) in other studies (Grendy et al, 1997). The K-*ras* gene encoded a protein of 21 kDa, p21, which were consisted of 188 amino acids (Santos and Nebreda, 1989). Ras protein as a critical relay switch that controls signalling pathways connecting the cell surface with the nucleus (Chen et al, 1996; Feliciello et al, 1996; Hughes et al, 1997). Ras protein is activated by growth factors, such as epidermal growth factor (EGF), insulin and insulin growth factor (IGF) (Leitner et al, 1997). It then triggers the activation of a cascade of serine/threonine kinase, which includes Raf-1 and MAPK (mitogen-activated protein kinase) (Porras and Santos, 1996). Activated MAPK then translocates to the nucleus where it phosphorylates and activates transcription factors which, in turn, cause changes in gene expression responsible for growth stimulation (Winston and Hunter, 1995). Like other guanine nucleotide-binding proteins, *ras* cycles between an active GTP-bound form and an inactive GDP-bound form. The GDP-bound form is converted to the GTP-bound form through a GDP/GTP exchange reaction that is facilitated by guanine nucleotide-releasing factors (Segal et al, 1993; Overbeck et al, 1995; Lin et al, 1996). On the other hand, the GTP-bound form is converted to the GDP-bound form by the intrinsic GTPase activity, which is accelerated by GTPase-activating proteins (GAPs) (Molloy et al, 1995; Miao et al, 1996; Wittinghofer et al, 1997). Since hydrolysis of GTP was an important event in regulation of p21 activity, it was suggested that mutation of *ras* gene might affect normal GTP hydrolysis. Numerous researchers have analysed the *ras* gene and reported that the mutation at codon 61 cause the mutant proteins to be unresponsive to the stimulation by the GTPase activating proteins, furthermore, increase the *ras*-transforming potential (Adari et al, 1988). However, other results showed that when a substitution occurred at codons 12, 13 or 59 of the *ras* gene, the Ras protein changed and furthermore lost its ability to bind with GTP (Barbacid, 1987). Consequently, mutant Ras persists in an active, GTP-bound state, leading to constitutive and deregulated stimulation of growth regulatory process that contributes to the aberrant growth properties of tumour cells (Downward, 1996). Our previous findings indicated that K-*ras* gene was mutated at codons 15, 16, 18 and 31. Because these mutations occurred frequently in the patients with adrenocortical tumours, we believe that they are associated with the development of adrenal tumours. Therefore, it is important to know whether these mutations affect the GTPase activity of Ras in the presence or absence of GAP to understand the mechanism of the carcinogenesis of adrenal tumours. In this way, we first examined the intrinsic GTPase activity of mutant

*Received 7 July 1999*
*Revised 7 October 1999*
*Accepted 18 October 1999*

*Correspondence to:* S-R Lin

**Table 1** K-*ras* gene expression constructs from human adrenocortical tumours and their gene products

| | | K-*ras* mutant | | |
|---|---|---|---|---|
| Plasmid | Vector | Codon base | Amino acid | Protein |
| pKWTCMV | pBKCMV | Wild type | | p21BN |
| pK1516MCMV | pBKCMV | 15GGC→ACA | Gly→Thr | |
| | | 16AAG→GAG | Lys→Glu | p21BM1 |
| | | 15GGC→ACG | Gly→Thr | |
| pK568MCMV | pBKCMV | 16AAG→AAA | Lys→Lys | |
| | | 18GCC→GTC | Ala→Val | p21BM2 |
| | | 31GAA→CAA | Glu→Gln | |
| pK60MCMV | pBKCMV | 60GGT→TGT | Gly→Cys | p21BM3 |

K-Ras protein ($p21^{K\text{-}Ras}$) and its GTPase activity in the GAP presence. We hope the results can provide a further understanding of the correlation between K-*ras* oncogene and adrenal tumours.

### MATERIALS AND METHODS

### Construction of mutant K-*ras* expression plasmids mRNA was extracted from tumour tissue and reverse transcribed (RT) to cDNA with MMLV reverse transcriptase and oligo (dT). Then, cDNA was amplified by polymerase chain reaction (PCR) with primers specific for coding region and 5′ and 3′ non-coding region of K-*ras* including *Kpn*I and *Sac*II site as follows: 5′-TTGGTACCCCTAAAAATGACTGAATATAAACTTGT-3′ and 5′-CGAGCTCGACCACTTGTACTAGTATGCC-3′. A 615 bp PCR product was amplified and inserted into the vectors pBKCMV (Stratagen, La Jolla, CA, USA) to prepare K-*ras* gene expression constructs for prokaryotic and eukaryotic cells. For prokaryotic cells, the vector pBKCMV was used. This vector contained a lac promoter which could regulate K-*ras* gene expression in *Escherichia coli*. In our experiments, wild-type and mutant K-*ras* genes, including mutations at codons 15, 16, 18 and 60, separately were cloned into the pBKCMV vector and named as pKWTCMV, pK1516MCMV, pK568MCMV, pK60MCMV (Table 1).

### Purification of mutant K-*ras* protein synthesized by *E. coli*

The bacterially synthesized K-Ras protein was purified as described earlier (Gross et al, 1985). Ten grams of *E. coli* cells were sonicated in 50 mM Tris–HCl, pH 8.0, 5 mM EDTA, 1 mM phenylmethylsulphonyl fluoride (PMSF) (5 ml $g^{-1}$ of cells), and the sonicated cell extract was then centrifuged for 10 min at 4000 ***g*** to remove unbroken cells and cell debris. The particulate fraction was pelleted by ultracentrifugation for 2 h at 3000 rpm. The pellet was homogenized in an excess of buffer A (50 mM Tris–HCl, pH 7.4, 5 mM 2-mercaptoethanol, 1 mM EDTA, 1 mM PMSF) and the suspension was centrifuged as above. The pellet was suspended in buffer A (2 ml $g^{-1}$ of cells), and Ras protein was solubilized by the addition of an equal volume of 7 M quanidine-HCl. The clear supernatant containing Ras protein was obtained after centrifugation for 2 h at 30 000 rpm and stored at –20°C before use.

### Construction of GAP expression plasmid

Using the PCR we constructed the expression plasmid for minimal fragments of $p120^{GAP}$: GAP-273 (residues $Met^{714}$ – $His^{986}$) from the bovine brain cDNA. The oligonucleotides used were as follows: 5′-CCGGAATTCCCGACCCCGCCGACCCCGATGGAAAAAATCATGCC-3′ and 5′-AGCGGCCGCTTTATTAATGCTCT-GTAGTGTCCGC-3′. PCR products were isolated, digested with *Eco*RI/*Not* I and ligated into the GST fusion vactor pGEX-4T-2 (Amersham, Pharmacia Biotech UK Ltd). The resulting plasmid, pGEX-GAP, was transformed into *E. coli* strain TG1. The correct orientation of the GAP cDNA was confirmed by sequencing.

### Expression, purification and cleavage of GST-GAP fusion protein

The expression of fusion protein was performed basically as described by Guan and Dixon (1991). Twenty millilitres of an overnight culture was inoculated into 1 litre of 2XYT (10 g yeast extract, 16 g tryptone and 5 g sodium chloride (NaCl) in 1 litre) containing 50 μg $ml^{-1}$ ampicillin. The culture was incubated vigorously at 37°C until an absorption of one at 600 nm was reached. IPTG was then added and cells were harvested by centrifugation and suspended in 10 ml PBST (2 mM EDTA, 0.1% β-mercaptoethanol, 0.2 mM PMSF and 5 mM benzamidine) after 3 h incubation. Cells were lysed and bacterial lysate was centrifuged at 4°C to remove the insoluble fraction. One millilitre of the bacterial supernatant containing soluble proteins was mixed with 2 ml 50% (v/v) glutathione agarose beads and incubated 30 min at 4°C with gentle shaking. The agarose beads were washed four times with 10 ml PBST. The fusion protein was eluted by competition with glutathione using 2 × 2 min washes with 1 ml of 50 mM Tris (pH 8.0) containing 10 mM glutathione. Free glutathione was removed by dialysis against 50 mM Tris (pH 8.0), 150 mM NaCl, 0.1% β-mercaptoethanol. Approximately 2-mg fusion protein was incubated with 4 μg of thrombin at room temperature for 20 min in 2 ml of thrombin cleavage buffer (50 mM Tris, (pH 8.0), 150 mM NaCl, 2.5 mM calcium chloride ($CaCl_2$), 0.1% β-mercaptoethanol). Glutathione S-transferase and uncleaved fusion protein were removed by directly adding 2 ml of glutathione agarose (50% v/v) to the cleavage reaction. The sample was incubated for 30 min at 4°C and centrifuged for 10 min. The supernatant, which contained purified GAP-273 protein was recovered. GTPase activating activity of GAP-273 was assayed by adding H-Ras (Santa Cruz Biotechnology, Inc) expressed in *E. coli*.

### Immunoblot

K-Ras protein from *E. coli* transformed with K-*ras* expression plasmids was purified and electrophoresed through vertical slab gels composed of 10% polyacrylamide containing 0.1% sodium dodecyl sulphate (SDS) prepared in 3 mM Tris–HCl, pH 8.8. Following electrophoresis, the protein was electroblotted to a nitrocellulose membrane (Schleicher & Schusll GmbH, Dassel, Germany). The membrane fixed with protein was incubated with mouse monoclonal antibodies (mAbs) specific for the K-Ras p21 product (25 mg $ml^{-1}$) (Santa Cruz Biotechnology, Inc.), and the excess unbounded antibodies were then washed off. The membrane binding with the mouse mAbs was incubated with a solution of secondary goat anti-mouse IgG conjugated with alkaline phosphate (1:3000, Bio-Rad, Hercules, CA, USA). The

 © 2000 Cancer Research Campaign tagged Ras protein was detected by enhanced chemiluminescence (ECL) detection system (Amersham, Pharmacia Biotech UK Ltd). Blots was then exposed to Kodak XAR film (Eastman Kodak, Rochester, NY, USA).

### Measurement of GAP activity

GAP assay was measured as described by Li et al (19). Two picomoles of Ha-Ras-[$\gamma$-$^{32}$P]GTP (30 Ci mol$^{-1}$, ICN) was incubated at 30°C with the indicated amounts of GAP-273 in 20 µl of a solution containing 50 mM Tris–HCl (pH 8.0), 5 mM magnesium chloride ($MgCl_2$), 0.25 mM GTP, 5 mg ml$^{-1}$ bovine serum albumin and 5% glycerol. The reactions were quenched by keeping the sample on ice. After the addition 0.5 ml of ice-cold washing buffer (20 mM Tris–HCl (pH 8.0), 100 mM NaCl, 5 mM $MgCl_2$), the sample was passed through a nitrocellulose filter (0.45 µm, Schleicher & Schusll GmbH, Dassel, Germany). The filter was washed three times with 1 ml of ice-cold washing buffer, and the radioactivity trapped on the filter was determined using a liquid scintillation counter. The amount of GTP hydrolysed during the reaction was determined as a decrease in the reactivity trapped on the filter. The data were calculated from at least three independent experiments.

### GTPase assay

Purification K-Ras protein (0.8 µg) was incubated with 60 µg of K-Ras p21 mAb (Santa Cruz Biotechnology, Inc.) in 100 µl of GTP buffer containing 0.1% Triton X-100, 50 µM PMSF and 0.3 mg ml$^{-1}$ BSA at 0°C for 60 min. Then, 2 µl of protein A-Sepharose beads (Amersham, Pharmacia Biotech UK Ltd), coated with rabbit anti-rat IgG and equilibrated in GTP buffer/0.1% Triton, was added and the sample was mixed for 25 min at 4°C. The beads were washed extremely with GTP buffer containing 0.12 M $(NH_4)_2SO_4$ and 0.1 mg ml$^{-1}$ BSA. The beads were then resuspended in 120 µl of this latter buffer containing 25 µM [$\alpha$-$^{32}$P]-GTP (15 $TB_{eq}$ mmol$^{-1}$; Amersham, Pharmacia Biotech UK Ltd) activity was measured by removing sample of the beads at each time, washing in GTP buffer/0.01% Triton and elution at 65°C with EDTA–SDS then layer chromatography (TLC) sample buffer. TLC analysis was performed as described below. To monitor GTPase activity stimulated by GAP. Twenty-five microlitres of the solution containing (8,5′-3H) $\alpha$-$^{32}$P-GTP-bound *Ras* protein was incubated with appropriate amount of GAP-273 at 30°C. At different time, 5 µl of the mixture was mixed taken to with 5 µl 0.5% SDS, 10 mM EDTA, 100 mM GTP and 100 mM GDP and taken heated to 80°C for 5 min. The mixture was immediately blotted on PEI-cellulose TLC plates and chromatographed in 0.5 M lithium chloride and 1 M formic acid solution. GTP and GDP spots were then cut off from the TLC plate separately and the radioactivity was determined. The rate of GTP hydrolysis was obtained from semilogarthnic plotting of the amount of bound GTP against time.

### RESULTS

The translational products of mutant K-*ras* gene constructs from human adrenocortical tumours made by *E. coli*, were showed in the 12.5% SDS polyacrylamide gel in Figure 1A. Then, 3.5 M guanidine–HCl was used to extract the K-Ras protein from the washed particulate fraction and a few other proteins might also be extracted. However, the intensity of the bands in Figure 1 showed that 85% of the proteins were K-Ras proteins. Further immunoblot analysis proved that those proteins could interact with mAbs specific for K-Ras p21 proteins, confirming that they were p21$^{K\text{-}Ras}$ proteins (Figure 1B). Comparison with the M.W. marker revealed that these K-Ras protein were 23 000 dalton.

The 273-residue fragment from p120$^{GAP}$ from Met$^{714}$ to His$^{986}$ could be expressed in *E. coli* as a glutathione S-transferase (GST) fusion protein. Figure 2 shows that the protein was isolated as GST fusion protein and cleaved from GST by thrombin. It remained soluble after purification. The specific activity of GAP-273 for

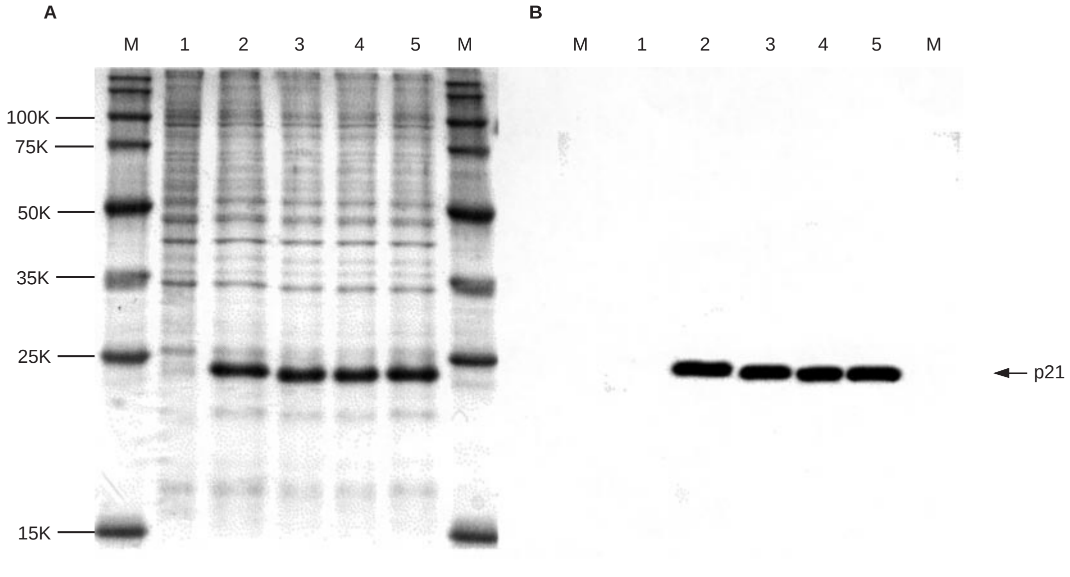

**Figure 1** SDS-PAGE and Western blot analysis of the translational products of mutant K-*ras* gene constructs from human adrenocortical tumours made by *E. coli.* K-Ras protein from *E. coli* transformed with pBKCMV (lane 1), pKWTCMV (lane 2), pK1516MCMV (lane 3), pK568MCMV (lane 4) or pK60MCMV (lane 5) were separated on a 10% polyacrylamide gel stained with Coomassie blue (**A**). The gel was followed by electroblotting to a nitrocellulose membrane. The blotted filter was probed with monoclonal antibody to K-Ras p21 protein. The filter were probed with a secondary alkaline phosphotase-conjugated goat anti-mouse IgG. The tagged K-Ras protein was detected by ECL detection system, then exposed to Kodak XAR film (**B**). Lane M, molecular weight standard

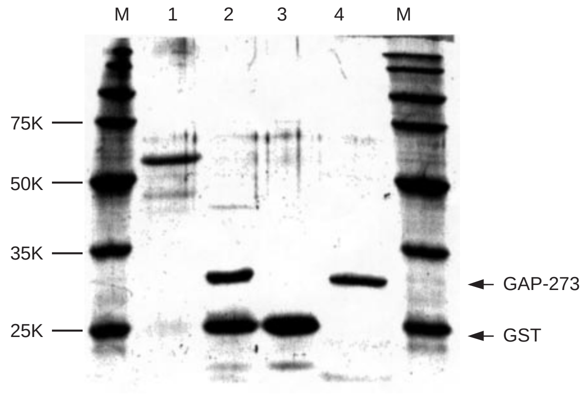

**Figure 2** Expression of the minimal fragment of p120[GAP]. GAP-273 were expressed as GST fusion protein using improved GST expression vector and purified as seen here in an SDS-polyacrylamide gel stained with Coomassie blue. Protein in lane 1 was the uncleaved 56-kDa GST-GAP fusion protein. Lane 2 represents the reaction mixture of GST–GAP fusion protein after thrombin cleavage. The GAP-273 (30 kDa) in lane 3 and glutathione S-transferase (26 kDa) in lane 4 are major components of the reaction mixture

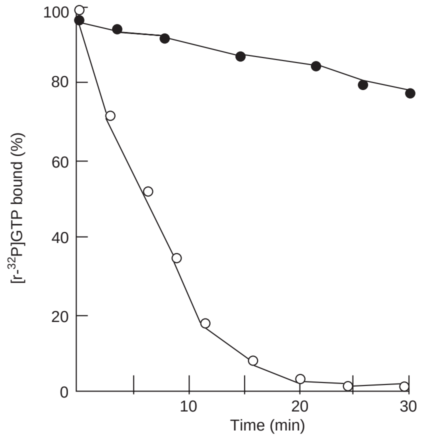

**Figure 3** Time course of the GAP-273-stimulated GTP hydrolysis by H-Ras protein. Two picomols of Ha-Ras-[γ-$^{32}$P]GTP were incubated at 30°C for indicated periods of time with (open symbols) or without (filled symbols) GAP-273. The reaction mixture was passed through a nitrocellulose filter. The filter was washed and the radioactivity trapped on the filter was determined using a liquid scintillation counter. The amount of GTP hydrolysed was determined as a decrease in the reactivity trapped on the filter. The data are shown as relative percentages catalysed hydrolysis of Ha-Ras GTP was shown in Figure 3. In Figure 3, GAP-273 purified from GST fusion protein is actually able to catalyse the hydrolysis of Ras GTP. Therefore, we used this purified GAP-273 protein to regulate the GTPase activity of mutant K-Ras proteins in our experiments.

The GTPase activities of these K-Ras proteins were measured both in the absence and presence of GAP-273. When the four different K-Ras proteins were analysed for their intrinsic GTPase activity, we found that the difference of K-Ras proteins of similar purity and quantity were shown in GTPase reaction, however, they have very high reproductivity. p21BN K-Ras protein, which is the wild-type K-Ras protein, reacted very similarly to p21BM1 and p21BM2 in intrinsic GTPase activity in which about 20% of the substrate GTP is hydrolysed by p21BN protein after 1.5 h (Figure 4). However, GTPase reactions of p21BM3 were 10 times lower than that of p21BN (Figure 5).

K-Ras mutant protein, p21BM3 and wild-type p21BN were purified with the same procedure and analysed by immunoprecipitation of intrinsic GTPase activity, suggesting that they should have similar purity and the same experimental errors. However, the GTPase activity of the two proteins were 8–15 times different. Therefore, we thought that these GTPase reaction were actually caused by K-Ras protein. In addition, other recombinant protein of similar size made in *E. coli* under the control of the same expression plasmid and solubilized from the particulate fraction of bacterial cell extracts in an identical manner to that used for K-Ras protein had minor contaminants similar to those in the K-Ras protein preparations yet exhibited no trace of GTPase activity. The above results indicated that the difference in GTPase activity was actually caused by purified K-Ras protein, only the K-Ras protein mutated at codon 60 from human adrenocortical tumour influenced the intrinsic GTPase activity.

In the GTPase activity assay with the GAP stimulation, the mutant p21BM1 K-Ras protein mutated at codon 15 and 16, in both the absence and presence of purified GAP-273 protein, it had the same GTPase activity as wild-type p21BN K-Ras protein. Although the intrinsic GTPase activity of p21BM3 K-Ras protein, which mutated at codon 60, decreased the intrinsic GTPase activity, the GAP-stimulated GAPase activity of this mutant protein was similar to that of the wild-type K-Ras protein. By contract, the mutant K-Ras protein, which mutated at codons 15, 16, 18 and 31, had the same intrinsic GTPase activity to this of wild-type K-Ras protein, but their GTPase activity was significantly lower than wild-type K-Ras protein after stimulation with GAP. Thus, we concluded that of the mutant K-Ras proteins from human adrenocortical tumours, only the mutant K-Ras protein which mutated at codons 15, 16, 18 and 31, influence GAP-sensitivity.

### DISCUSSION

The K-*ras* gene has two alternative 3′ exons designated 4A and 4B (Capon et al, 1983). p21$^{K\text{-}rasA}$ and p21$^{K\text{-}rasB}$ are similar but different in the 24/25 C terminal residues. Numerous reports have shown that K-*ras*B appears to be ubiquitously expressed (Slamon and Cline, 1984; Leon et al, 1987); in contrast, the expression of K-*ras*A is induced during differentiation of pluripotent embryonal stem cells in vitro (Pells et al, 1997). In addition, our previous study of human adrenocortical tumours showed that mutant and expressed K-*ras* genes are mainly K-*ras*B gene. Therefore, we cloned K-*ras*B cDNA from tumour cells into a vector and then transformed the K-*ras* gene constructs into *E. coli*. The K-Ras proteins expressed in *E. coli* transformed with K-*ras* gene constructs were observed through SDS-gel electrophoresis. The results showed that these K-Ras proteins migrate with an apparent molecular weight slightly larger than that of natural p21$^{Ras}$ protein. This is probably due to the inability of *E. coli* to carry out the post-translational modification with p21$^{Ras}$ undergoes in mammalian cells (Lantenberger et al, 1983).

In the cell the conformational state of guanine nucleotide binding proteins is regulated by two kinds of interacting molecules, guanine nucleotide exchange factors and GTPase activating proteins (called GAPs). Three specific GAPs for p21$^{Ras}$ were p120$^{GAP}$ (the prototype of this class of proteins and the first one to be isolated) (Vogel et al, 1988; Lim et al, 1992), neurofibromin (NF1) (Hattori et al, 1992) and a mammalian homologue (GAP1$^{m}$)

*© 2000 Cancer Research Campaign*

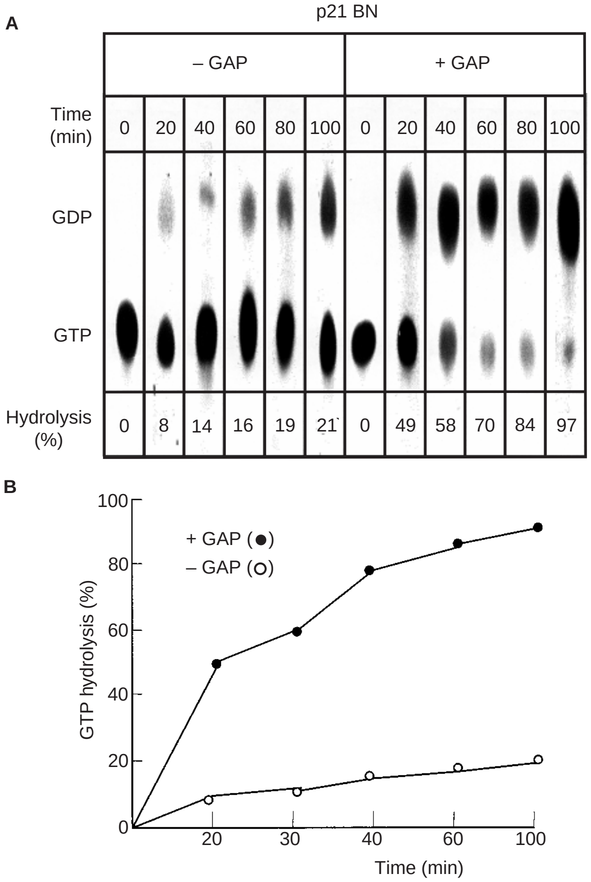

**Figure 4** Time course of the GAP-273-stimulated GTP hydrolysis of wild-type K-Ras protein, p21BN. Shown is the GTP hydrolysis reaction catalysed by recombinant K-Ras protein, p21BN in the absence and presence of GAP-273. The reaction was conducted at 30°C and samples were taken at the indicated times. The reaction products were analysed by TLC, followed by autoradiography (**A**) and quantification of the radioactive GTP and GDP spots using a PhosphoImager (Molecular Dynamics) (**B**)

of the Drosophilic GAP1. p120$^{GAP}$ and NF1 protein can be distinguished with respect to their catalytic properties (Martin et al, 1990; Xu et al, 1990; Nur-E-Kamal and Maruta, 1992). p120$^{GAP}$ increases the GTPase reaction of p21$^{Ha\text{-}Ras}$ more than tenfold catalytic activity for Ras GTP, whereas NF1 protein has been reported to have a lower activity but a higher affinity. In this study, we demonstrated that GAP-273 (codons 174–986) could stimulate the GTP hydrolysis of Ha-Ras protein. This is consisted with the result made by Ahmadian et al (1996). In 1996, Ahmadian et al confirmed that the specific activity of GAP-273 for catalysed hydrolysis of Ras bound GTP was almost the same as that measured with full-length p120$^{GAP}$. Therefore, we used GAP-273 as the GAP of mutant K-Ras in this study.

Regarding the analysis of GTPase activity and GAP sensitivity, the results showed that only mutation at codon 60 influenced intrinsic GTPase activity. This may be due to the fact that codon 60 (Gly) is the only amino acid without a branch among the amino acids on phosphate binding region (codons 57–63) (Sung et al, 1995). In the mutant K-Ras protein, the cysteine substitution caused this amino acid (Gly) to add a sulphur, which affected its conformation and furthermore affected its binding with GAP, then enhancing the activity of GTPase (Boriack-Sjodin et al, 1998). The same phenomena was also observed in the K-Ras protein with mutation at codon 28 (Powers et al, 1989). On the other hand, the mutant K-Ras protein with mutation at codons 15, 16, 18 and 31 had lower GAP sensitivity than normal. These results can be explained by the reports of Sigal et al (1986), Powers et al (1989) and Shirouzu et al (1992). In 1986, Sigal et al found p21 protein Lys-16 was the decision site of the GTP/GDP-binding site. If Lys-16 is replaced by Asn, the affinity between GTP and GDP will decrease 100 times, without affecting GTP/GDP-binding specificity. In 1989, Powers et al designed a mutant p21 protein with Ala instead of Gly-15 for studying the characteristics of the p21 protein. The results showed that this alteration could also affect the normal functions of the p21 protein. In addition, Shirouzu et al (1992) found that Ras protein with Glu-31→Lys mutation was found to have drastically decreased GAP sensitivity. Thus they

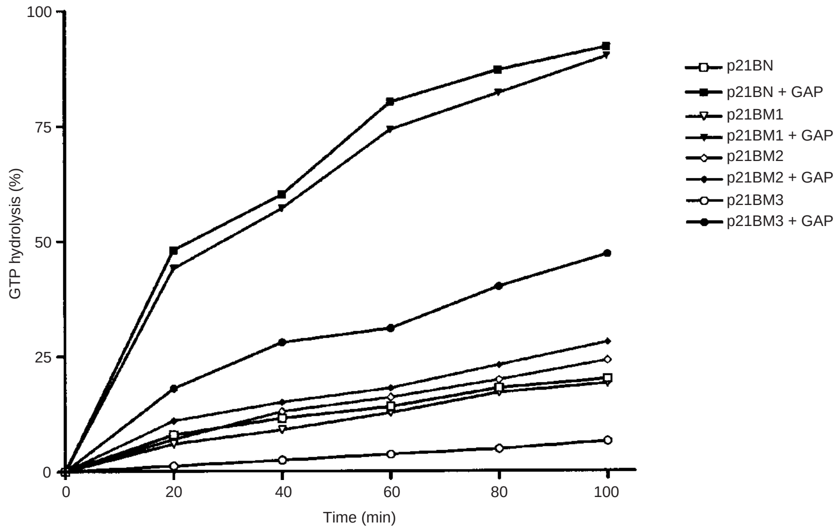

**Figure 5** Time course of the GAP-273-stimulated GTP hydrolysis of mutant K-Ras proteins. The GTPase activity was determined by 25 μl of the solution containing (8, 5′-3H) α-$^{32}$ P-GTP bound Ras protein was incubated with (filled symbol) or without (open symbol) GAP-273 at 30°C. At different time, 5 μl of the mixture was analysed by TLC. GTP and GDP spots were then cut off from the TLC plate separately and the radioactivity was determined. The rate of GTP hydrolysis was obtained from semilogarthnic plotting of the amount of bound GTP against time

*© 2000 Cancer Research Campaign*

concluded that p21 Ras protein Glu-31 is an important residue for the simulation of GTPase activity by GAP[Ras]. However, the K-Ras protein with mutation at codons 15 and 16 from another tumour did not reveal a decrease in GAP sensitivity. This may be caused by different amino acid substitution between the two mutant K-Ras protein.

In this study, we observed that mutant K-*ras* gene from human adrenocortical tumour has indeed decreased the GTPase activity of K-Ras protein. Our advanced study showed that when the normal adrenocortical cell transfected with mutant K-*ras* expression plasmids derived from human adrenocortical cells by electroporation, the levels of cortisol secreted by transfected cells were 15–25 times higher than normal cells (Lin et al, 1999). However, the mechanisms by which K-Ras protein affects the adrenocortical tumour development needs more study.

### ACKNOWLEDGEMENTS

This study was supported by a grant from the National Science Council of the ROC (NSC-83-0412-B037-030).

### REFERENCES

Adari H, Lowy DR, Willumsen BM, Der CJ and McCormick F (1988) Guanosine triphosphatase activating protein (GAP) interacts with the p21 *ras* effector binding domain. *Science* **240**: 518–521

Ahmadian MR, Wiesmuller L, Lautwein A, Bischoff FR and Wittinghofer A (1996) Structural differences in the minimal catalytic domains of the GTPase-activating proteins p120GAP and neurofibromin. *J Biol Chem* **271**: 16409–16415

Anker P, Lefort F, Vasioukhin V, Lyautey J, Lederrey C, Chen XQ, Stroun M, Mulcahy HE and Farthing MJ (1997) K-*ras* mutations are found in DNA extracted from the plasma of patients with colorectal cancer. *Gastroenterology* **112**: 1114–1120

Barbacid M (1987) *Ras* gene. *Ann Rev Biochem* 56: 779–827

Boriack-Sjodin PA, Margarit SM, Bar-Sagi D and Kuriyan J (1998) The structural basis of the activation of Ras by Sos [see comments]. *Nature* **394**: 337–343

Caduff RF, Svoboda-Newman SM, Ferguson AW, Johnston CM and Frank TS (1999) Comparison of mutations of Ki-Ras and p53 immunoreactivity in borderline and malignant epithelial ovarian tumors. *Am J Surg Pathol* **23**: 323–328

Capon DJ, Seeburg PH, McGrath JP, Hayflick JS, Edman U, Levinson AD and Goeddel DV (1983) Activation of Ki-*ras*2 gene in human colon and lung carcinomas by two different point mutations. *Nature* **304**: 507–513

Chen Q, Lin TH, Der CJ and Juliano RL (1996) Integrin-mediated activation of MEK and mitogen-activated protein kinase is independent of Ras. (Published erratum appears in *J Biol Chem* 1996 271: 22280.) *J Biol Chem* **271**: 18122–18127

Downward J (1996) Control of *ras* activation. *Cancer Surv* **27**: 87–100

Feliciello A, Giuliano P, Porcellini A, Garbi C, Obici S, Mele E, Angotti E, Grieco D, Amabile G, Cassano S, Li Y, Musti AM, Rubin CS, Gottesman ME and Avvedimento EV (1996) The v-Ki-*Ras* oncogene alters cAMP nuclear signaling by regulating the location and the expression of cAMP-dependent protein kinase II beta. *J Biol Chem* **271**: 25350–25359

Gao HG, Chen JK, Stewart J, Song B, Rayappa C, Whong WZ and Ong T (1997) Distribution of p53 and K-*ras* mutations in human lung cancer tissues. *Carcinogenesis* **18**: 473–478

Grendys EC Jr, Barnes WA, Weitzel J, Sparkowski J and Schlegel R (1997) Identification of H-, K-, and N-*ras* point mutations in stage IB cervical carcinoma. *Gynecol Oncol* **65**: 343–347

Gross M, Sweet RW, Sathe G, Yokoyama S, Fasano O, Goldfarb M, Wigler M and Rosenberg M (1985) Purification and characterization of human H-Ras proteins expressed in *Escherichia coli*. *Mol Cell Biol* **5**: 1015–1024

Guan KL and Dixon JE (1991) Eukaryotic proteins expressed in *Escherichia coli*: an improved thrombin cleavage and purification procedure of fusion proteins with glutathione S-transferase. *Anal Biochem* **192**: 262–267

Hattori S, Maekawa M and Nakamura S (1992) Identification of neurofibromatosis type I gene product as an insoluble GTPase-activating protein toward *ras* p21. *Oncogene* **7**: 481–485

Hughes PE, Renshaw MW, Pfaff M, Forsyth J, Keivens VM, Schwartz MA and Ginsberg MH (1997) Suppression of integrin activation: a novel function of a Ras/Raf-initiated MAP kinase pathway. *Cell* **88**: 521–530

Lautenberger JA, Ulsh L, Shih TY and Papas TS (1983) High level expression in *Escherichia coli* of enzymatically active Harvey murine sarcoma virus p21Ras protein. *Science* **221**: 858–860

Leitner JW, Kline T, Carel K, Goalstone M and Draznin B (1997) Hyperinsulinemia potentiates activation of p21Ras by growth factors. *Endocrinology* **138**: 2211–2214

Leon J, Guerrero I and Pellicer A (1987) Differential expression of the *ras* gene family in mice. *Mol Cell Biol* **7**: 1535–1540

Lim HH, Michael GJ, Smith P, Lim L and Hall C (1992) Developmental regulation and neuronal expression of the mRNA of rat n-chimaerin, a p21rac GAP:cDNA sequence. *Biochem J* **287**: 415–422

Lim L, Manser E, Leung T and Hall C (1996) Regulation of phosphorylation pathways by p21 GTPases. The p21 Ras-related Rho subfamily and its role in phosphorylation segralling pathways. *Eur J Biochem* **242**: 171–185

Lin SR, Tsai JH, Yang YC and Lee SC (1998) Mutations of K-*ras* oncogene in human adrenal tumors in Taiwan. *Br J Cancer* **77**: 1060–1065

Lin SR, Hsu CH, Tsai JH, Wang JY (1999) Effect of K-*ras* mutant derived from human primary aldosteronism on normal adrenocortical cells (submitted)

Martin GA, Viskochil D, Bollag G, McCabe PC, Crosier WJ, Haubruck H, Conroy L, Clark R, O'Connell P and Cawthon RM (1990) The GAP-related domain of the neurofibromatosis type 1 gene product interacts with *ras* p21. *Cell* **63**: 843–849

Miao W, Eichelberger L, Baker L and Marshall MS (1996) p120 Ras GTPase-activating protein interacts with Ras-GTP through specific conserved residues. *J Biol Chem* **271**: 15322–15322

Molloy DP, Owen D and Grand RJ (1995) Ras binding to a C-terminal region of GAP. *FEBS Lett* **368**: 297–303

Mulcahy HE, Lyautey J, Lederrey C, Chen X, Anker P, Alstead EM, Ballinger A, Farthing MJ and Stroun M (1998) A prospective study of K-*ras* mutations in the plasma of pancreatic cancer patients. *Clin Cancer Res* **4**: 271–275

Nur-E-Kamal MS and Maruta H (1992) The role of Gln61 and Glu63 of Ras GTPases in their activation by NF1 and Ras GAP. *Mol Biol Cell* **3**: 1437–1442

Overbeck AF, Brtva TR, Cox AD, Graham SM, Huff SY, Khosravi-Far R, Quilliam LA, Solski PA and Der CJ (1995) Guanine nucleotide exchange factors: activators of Ras superfamily proteins. *Mol Reprod Dev* **42**: 468–476

Pells S, Divjak M, Romanowski P, Impey H, Hawkins NJ, Clarke AR, Hooper ML and Williamson DJ (1997) Developmentally-regulated expression of murine K-*ras* isoforms. *Oncogene* **15**: 1781–1786

Porras A and Santos E (1996) The insulin/Ras pathway of adipocytic differentiation of 3T3 L1 cells: dissociation between Raf-1 kinase and the MAPK/RSK cascade. *Int J Obes Relat Metab Disord* **20**: S43–51

Powers S, O'Neill K and Wigler M (1989) Dominant yeast and mammalian Ras mutations that interfere with the CD23-dependent activation of wild-type RAS in *Saccharomyces cerevisiae*. *Mol Cell Biol* **9**: 390–395

Santos E and Nebreda AR (1989) Structural and functional properties of ras proteins. *FASEB J* **3**: 2151–2163

Segal M, Willumsen BM and Levitzki A (1993) Residues crucial for Ras interaction with GDP-GTP exchangers. *Proc Natl Acad Sci USA* **90**: 5564–5568

Shirozu M, Fujita-Yoshigaki J, Ito Y, Koide H, Nishimura S and Yokoyama S (1992) A glutamic acid residues at position 31 of Ras protein is essential to the signal transduction for neurite outgrowth of PC12 cells and the stimulation of GTPase activity by GAP[Ras]. *Oncogene* **7**: 475–480

Slamon DJ and Cline MJ (1984) Expression of cellular oncogenes binding embryonic and fetal development of the mouse. *Proc Natl Acad Sci USA*: 7141–7145

Sung YJ, Carter M, Zhong JM and Hwang YW (1995) Mutagenesis of the H-*Ras* p21 at glycine-60 residue disrupts GTP-induced conformational change. *Biochemistry* **34**: 3470–3477

Vogel US, Dixon RAF, Schaber MD, Diehl RE, Marshall MS, Scolnick EM, Signal IS and Gibbs JB (1988) Cloning of bovine GAP and its interaction with oncogenic *ras* p21. *Nature* **335**: 90–93

Winston LA and Hunter T (1995) JAK2, Ras, and Raf are required for activation of extracellular signal-regulated kinase/mitogen-activated protein kinase by growth hormone. *J Biol Chem* **270**: 30837–30840

Wittinghofer A, Scheffzek K and Ahmadian MR (1997) The interaction of Ras with GTPase-activating proteins. *FEBS Lett* **410**: 63–67

Xu GF, Lin B, Tanaka K, Dunn D, Wood D, Gesteland R, White R, Weiss R and Tamanoi F (1990) The catalytic domain of the neurofibromatosis type 1 gene product stimulates ras GTPase and complements ira mutants of *S. cerevisiae*. *Cell* **63**: 835–841

Yamashita N, Minamoto T, Ochiai A, Onda M and Esumi H (1995) Frequent and characteristic K-ras activation in aberrant crypt foci of colon. Is there preference among K-*ras* mutants for malignant progression? *Cancer* **75**: 1527–1533

*© 2000 Cancer Research Campaign*
